# Pediatric Cardiac Service Development Programs for Low- and Middle-Income Countries in Need of Improving or Initiating Local Services

**DOI:** 10.3389/fped.2019.00359

**Published:** 2019-09-20

**Authors:** William M. Novick, Frank Molloy, Karen Bowtell, Brian Forsberg, Martina Pavanić, Igor Polivenok, Sri Rao, Yamile Muñoz, Marcelo Cardarelli

**Affiliations:** ^1^University of Tennessee Health Science Center, Global Surgery Institute, Memphis, TN, United States; ^2^William Novick Global Cardiac Alliance, Memphis, TN, United States; ^3^Great Ormond Street Hospital, London, United Kingdom; ^4^Children's Memorial Hermann Hospital, Houston, TX, United States; ^5^Zaitcev Institute for General and Urgent Surgery, Kharkiv, Ukraine; ^6^Department of Pediatrics, University of South Carolina School of Medicine, Columbia, SC, United States; ^7^Clinica Cardiovid, Medellin, Colombia; ^8^Department of Surgery, Inova Children's Hospital, Falls Church, VA, United States

**Keywords:** global surgery, humanitarian assistance, pediatric cardiac surgery, congenital heart, education

## Abstract

Pediatric cardiac services are deficient in most of the world. Various estimates are that between 80 and 90% of the world's children do not receive adequate cardiac care for their congenital or acquired heart disease. We began a modest effort in 1992 to assist in the development of pediatric cardiac services in low- and middle-Income countries (LMIC). Since then, we have provided services in 32 countries based on 3 distinctive development strategies, in order to meet the local needs for pediatric cardiac services. Our goal has always been to provide education, training and sufficient experience so that eventually we leave a site with a fully functional, independently operating pediatric cardiac service that is sustainable over time. The margin between success and failure is dependent upon a number of factors and we hope that this chapter will provide others with the tools for success.

## Introduction

Pediatric cardiac services are widespread in North America, Europe and along the industrialized portion of the Pacific Rim. However, in the remaining parts of the world these services range from simply inadequate to meet the national, regional or local need to entirely absent (1). The result is that an estimated 80–90% of the children in the world with various forms of heart disease do not have adequate care (2). The most common congenital defect, congenital heart disease (CHD), and a preventable disease, rheumatic fever, account for a significant proportion of the burden of child mortality in LMIC. Out of the over 1 million children born annually with CHD (3) 250,000 died untreated in the first year of life in those countries. Likewise, nearly 275,000 die of the cardiac complications of rheumatic fever (4). While industrialized nations have a very low surgical mortality for CHD or fully prevent rheumatic fever, these heart diseases continue to exert a taxing toll in children born in LMIC.

Pediatric cardiac surgery is relatively a young specialty, with a simple yet significant first successful ligation of a ductus arteriosus by Gross at Boston Children's Hospital in 1938. Followed 6 years later by Crafoord's first coarctation repair and Blalock-Taussig systemic to pulmonary artery shunt historical operations (5–7). Cardiopulmonary bypass followed years later after Lillehei had used cross-circulation to perform intra-cardiac surgery (8). By the late 50's only two centers in the United States (University of Minnesota and Mayo Clinic) and less than a handful in the world (9, 10) were performing open heart surgery and the spread of pediatric cardiac surgery did not began in earnest until the early 60's. We may struggle to understand why modern pediatric cardiac surgery, after being practiced and refined for 60 years has failed, unlike many other modern medical advances, to spread world-wide (11). Several issues come to mind including, but not limited to, excessive costs of imported capital equipment and disposables, resulting in a limited number of sites performing enough surgeries as to function as training sites. That combined with a much slower pace of communications so that new techniques and developments required months and sometimes years to spread within the scientific community. The result was that while centers developed and multiplied in the developed world, only but a few successful ones developed in LMIC. Assistance in pediatric cardiac surgery by teams from developed countries began in the 70's and has mushroomed since then (12–15). We began our assistance in Colombia in 1991 and since then have visited 32 countries, having completed over 560 trips of between 1- and 4-week duration, with nearly 10,000 patients operated to this date (16, 17). We have used a number of training-education models to develop sustainable programs, whether *de novo* or for improving existing sites. The success or failure of an assistance program hinges on multiple factors which we will explain in this chapter.

## Request for Assistance

The request for assistance (RFA) can come for practically any source. Over the years we have received RFA from; colleagues returning home after training, non-medical and medical ex pats living in the United States, pediatricians and pediatric cardiologists/surgeons in LMIC, civic groups/foundations in the United States or abroad, Hospital Directors and Ministers of Health (MOH) in LMIC and occasionally from the offices of executive power officials (Vice Presidents or Presidents) of LMIC. We have entertained every request with a response to determine the viability of the RFA. The process of validating a RFA varies depending upon the origin of the request. If the RFA comes from a Hospital Director, MOH or the Executive offices of the government, then they require less validation since the decision and the funding to implement have usually been approved (18). Meanwhile RFA coming from other sources may require substantial work. Once we have determined the validity of the source of the request, we send a detailed questionnaire regarding the hospital facilities and a separate one regarding the medications available locally (Appendices 1 and 2) for the local team to complete. Photos are requested with the returned questionnaire and from this we determine if a site visit is warranted. The major issues to be considered before a site visit is made are; adequate infrastructure and accessibility of the hospital, local staff in all specialties to train, equipment needs (will need to reconfirm on site visit), locally available supplies and medications, philosophical support for program development from the local team, hospital administration, regional and national health directorates/ministries and local need for such a service. Invariably the media will become involved which one must remember can be good or bad for the local program if not handled appropriately (19).

## Site Visit

Occasionally a site visit is not necessary when adequate information, photographic documentation and the completeness and veracity of responses can be verified by a trusted local or expat, fluent in the local language and preferably with some medical background. We have been able to make a first surgical trip without a site vetting visit to approximately 20% of the more than 50 institutions we have assisted. Site visits, when needed before the first trip, should be conducted by a biomedical engineer, operating room individual (surgeon or nurse), ICU person (physician or nurse) and an executive administrator representing the visiting group. The basic requirements must be reviewed for completeness in all areas of infrastructure, equipment, power plant capabilities (and back up), personnel and support services (radiology, laboratory, blood bank) (20). The language of the visiting team can be problematic if the local language is not the same, so translators will need to be present if needed.

Discussions with all local stakeholders is critical as a thorough understanding of local enthusiasm, support for the program and motivation must be determined in all area's personnel. We have found that it is important to hold meetings with individual groups by area first before a final discussion with the decision makers. Local personnel are frequently anxious of sharing their honest feelings or opinions when faced with administrative or decision-making individuals present. Moreover, it is imperative that you understand from the local team if there is a real consensus on what type of program is desired. The visiting team should not impose its plan for the site on the local team, rather the visiting team must adhere to the local desires for their program's development. The plan must be consistent otherwise frustration can develop on both sides. Once a decision is made as to the type of program a site desires a plan can be drafted, a program can be implemented, and actions undertaken (21).

## Preparation for Program Implementation

Once the site visit is completed a comprehensive overview of the deficiencies in infrastructure, equipment, supplies, medications and personnel should be constructed in a concise yet complete manner. Depending upon the visiting teams experience in providing pediatric cardiac services in LMIC this may be done before departure or upon returning home. The benefit to preparing the assessment before departure is that it can be discussed with the local stakeholders prior to the site visit team's departure and earlier decisions made regarding who will be responsible for correcting or alleviating the deficiencies, thus allowing both sides to develop a financial budget for the program (22). Funding is a critical issue and the visiting team should have a thorough understanding of who will be responsible for each line item in the budget needed to correct deficiencies, transport the visiting team and house them on location. The total expenses for a program will depend upon the correction of the deficiencies as well as the number of trips to be made within the timeframe of the program.

## Program Models

We are committed to the initial visiting team model as a means to assess the local team and to determine which of our teaching/training model will provide the local team with the result, they desire upon program completion. Essential in the assessment is a discussion with the local stakeholders regarding the length of time they want to achieve the final result of an independently functioning pediatric cardiac service or improved service. Requests vary for program endpoints, from a service able to provide routine pediatric cardiac surgery to those wishing to improve their neonatal results. There are models used by others which have been successful, but our focus is on the efforts we have employed over the last 27 years.

### Intermittent Visit Model

The Intermittent Visit Model (IVM) is the most frequently used model by us as well as other charities to provide clinical and educational services (23–25). In this model a team of pediatric cardiac specialists visits the LMIC hospital for a variable period of between 1 and 4 weeks. Early in the program, our teams tend to be larger with enough individuals to provide safe and complete care to all children undergoing intervention (catheterization or surgery). Our usual team composition consists of senior pediatric cardiac surgeon, pediatric cardiac anesthesiologist, pediatric perfusionist, scrub nurse, pediatric cardiologist, pediatric intensivists, pediatric cardiac ICU nurses, pediatric respiratory therapist and biomedical engineer. Total team size is dictated by the number of ICU beds and operating rooms available. The team works as the primary caregiver until we determine the capabilities of the local staff who are working side by side with all our team members from the start. We are adherents to the concept of graduated responsibility based upon the local individuals' skills and capability. Moreover, we are advocates of high-volume caseloads since multiple publications have shown that large volume centers tend to have lower mortality rates (26, 27). Literature in surgical education suggests that the more the trainee operates in simulations the faster and thoroughly they learn surgical skills (28). Simulation is impractical for cardiac surgical trainees in most LMIC secondary to cultural issues and so volume of cases performed is important (29). Certainly, the same applies across all specialties involved in pediatric cardiac care. We must caution however on only operating for large volumes of cases, whether in the OR or Cath lab. An ICU filled to capacity with high acuity patients with a limited number of experienced staff can lead to unwanted events and mistakes (30–32).

Under optimal circumstances and with appropriate severity case scheduling, up to 16–20 children may receive surgery during a 2-week visit. We do not like to begin any program at an inexperienced site with neonatal operations (33). Ideally, we begin each trip with RACHS-1 category I and II cases and only once we are certain that all organizational, personnel and equipment issues are solved we will move to category III and IV cases (34). We have started all our programs in this manner when working at either inexperienced or *de novo* program. Sites with developed programs requesting assistance to help improve care with complex cases or neonatal surgery are started much differently as dictated by the requested need.

The frequency of annual visits and the duration of the program are dependent upon the end-point the institution desires to achieve the time they want to achieve the goal and of course the available funding for supporting the endeavor. Our experience is that with *de novo* programs 5–7 years may be required with 3–4 visits annually. Programs that are established require less time, but we believe the frequency of visits should not fall below 2 annually. Less visits allow a return to the old habits and standards that you have been asked to change.

Mentorship in every aspects of the specialty is the cornerstone of our educational approach. However, scheduled and impromptu lectures and learning opportunities constitute a significant part of the educational experience we provide (35). Superb educational opportunities exist in two very important multi-disciplinary meetings: The patient evaluation/management conference (held at the beginning of a trip) and the daily morning rounds in the ICU (36, 37). The cardiology/cardiac surgery management conference is based upon the pre-visit evaluations and diagnostic studies performed by the local caregivers. All specialties are invited to attend, and interactive participation is encouraged and expected and occasionally solicited from the local participants who are not cardiologists. Expected participants aside from the surgeons and cardiologists are anesthesiologists, perfusionists and the lead ICU nurses from the visiting and local teams. Each case presented is thoroughly reviewed and if additional studies are required an explanation is provided. A surgical approach is recommended by the lead visiting surgeon and options solicited from the local team. Once the cases are reviewed and plans agreed to, then a surgical schedule is constructed that will provide optimal movement and bed availability in the ICU. Emergencies can alter the schedule and we leave the decision of which case to cancel so the emergency can be cared for to the local team. Daily multi-disciplinary rounds are made in the ICU each morning. We provide 24-h ICU coverage with our team's ICU nurses and either an Intensivist or Nurse Practitioner. Morning rounds in the ICU are led by the bedside nurse who cared for the child during the night. Upon completion of the trip the afternoon of the last day is reserved for the debriefing session.

### Resident Senior Surgeon Model

A senior retired surgeon or a surgeon fully dedicated to humanitarian efforts can assume residence in-country for several years or permanently (38). This model generally occurs at a site where an existing program wishes to expand surgical volume and/or complexity and often to improve morbidity and mortality. We have no experience in using this model to establish *de novo* programs. We have used this model successfully in Nicaragua. Several years are required, and the surgeon-in-residence must be prepared to provide guidance in all aspects of pediatric cardiac services; clinical, administrative, financial, media relations and politics. The growth of the assisted program requires careful yet progressive nurturing and the support of the hospital administration. The development of a relationship with a local charity and with philanthropic individuals and corporate entities is critical to the success of the program.

The clinical management is similar to the Intermittent Visit Model but due to the number of inexperienced local healthcare professionals the volume of work is usually less initially. Education is at all levels and the establishment of locally driven continuing medical education is a must. A Quality Improvement program should be started, and monthly meetings held with all stakeholders involved in the clinical care. Research projects can be generated and should be pursued at both the nursing and medical staff levels. The initial design and implementation of a patient registry and database provide the site with the opportunity to review results regularly, identify problems early, provide data for clinical studies and stimulate patient follow-up. A relentless approach is necessary to achieve success with all development programs, but with this model it is essential that early in the process the surgeon-in-residence must enlist local leaders to cover all needs.

### Team in Residence Model

A very ambitious model is the Team in Residence (TIR) model. The concept is to place a team of specialists in-country residence for periods of 1–2 years on a nearly continuous basis (39). We have essentially 3 different but interconnected teams with this model; surgical team (surgeon, anesthesiologist, perfusionist, ± scrub nurse), the ICU team [(one or two Intensivists, a ICU Nurse Practitioner and/or Nurse Educator and 2 ICU nurses) and the cardiology team (interventional cardiologist]. The surgical team is on-site for 1 month and the ICU team for that month and then a week stay over, whereupon the surgical team returns with a new ICU team and the cycle repeats for the entire 12 months. Breaks occur according to the local and visiting team holiday calendar. Usually the surgical team is on-site 36–38 weeks out of 52 and the ICU team 44–46 weeks during the same period. The interventional cardiology team provides coverage when devices are available, and this usually is not more than 1–2 weeks every 3–4 months.

The major advantage to this model is that the local team is exposed and trained on a variety of cases from simple to complex with moderate volumes of patients each month (Table 1). The progress of the local team is usually faster by gaining responsibility as primary caregivers over a shorter period (40–44). The major disadvantages to this program are the expense and the difficulties regarding recruitment for the visiting team. We have used this model in only two countries, Iraq and Libya, but at 4 different sites to date (Nasiriyah in Iraq and Benghazi, Tobruk and Tripoli in Libya) (45, 46). Placing pediatric cardiac specialists in-country for 4–5 weeks could potentially be solved by a volunteer team, but not 9 times in 12 months. We have full time staff working for the foundation in each aspect of the specialty and they provide a steady foundation as the basic team. We augment the team with volunteer nurses and occasionally respiratory therapist in addition to the interventional cardiologist. Expenses are provided by the government of the country requesting help, usually from the Ministry of Health, but in some cases from the regional health care directorate.

**Table 1:** Variety and Complexity of Cases under Team in Residence Model.

	**Nasiriyah, Iraq** **(01/05/14–01/29/14)**	**Tripoli, Libya** **(06/04/18–06/29/18)**
**Age** ***(N)***		
Newborns	2	2
Infant	11	4
Children	30	34
Adults	2	0
Total	45	40
**Rachs Class**		
RACHS I	7	8
RACHS II	26	20
RACHS III	8	11
RACHS IV	1	1
Non-RACHS	3	0
Total	45	40

### Nursing Education/Empowerment

The process of collaboration to develop nurse education requires recognition of the already the established processes and practices at any given site. It is not conducive to building trust to come in and to “takeover.” Our organization focus on inclusion of the local team, respect for their ways and developing a practice over time. Our goal is to build sustainable services and this process requires that our local counterparts are invested and feel valued in the program.

Our philosophy is to educate the local nurses so that they can competently care for all the children before or after cardiac surgery or intervention. Nursing education in LMIC varies greatly and a thorough assessment of the educational foundation and experience in caring for children is necessary before clinical services begin (47). The formal learning needs assessment is performed on site during the first visit. During this stage each local nurse is directly supervised and supported by a nurse mentor from NCA. The direct supervision and support in the early phase enables us to provide education and explore local policies and procedures without compromising patient safety or quality of care.

The aim of nurse education is to ultimately empower local nurses to make decisions about the care they provide using a systematic process. Initial teaching focuses on task and skill acquisition, for example drug calculations, basic life support, equipment use, blood port sampling and recording of vital signs. With each subsequent visit by the team we reinforce the already learned practice and build the complexity of the educational development (48). In most of the countries we visit the nurse education system has focused on didactic teaching and memorization type learning which means the nursing team often “know” information, however, they have no experience of applying the principles of knowledge to different situations. Learning clinical decision making requires a different type of mental activity than merely remembering or knowing information and to facilitate this we introduce learning strategies that encourage productive models of thinking such as medium fidelity simulation and problem-based learning. These teaching modalities support the nurses as they learn to apply their new level of knowledge and reasoning to multiple clinical situations (49).

The ability to make decisions in practice is often aided by strong and supportive Multi-disciplinary team (MDT) working and we encourage each site to develop a flat hierarchy which empowers team members to contribute their ideas and opinions. In the ICU we undertake formal MDT ward rounds every day and nurse led handover is encouraged and facilitated. We actively perform this process from the first visit so that local nurses come to understand that they are active participants in the care of the children and that their contribution is essential in the child's recovery. The combination of education and empowerment of the nursing team aids in the development of nurses who can make informed and accurate clinical decisions, quickly detect patient deterioration and positively impact patient care (50).

### Interventional Cardiology Services

The addition of an interventional cardiology program constitutes a necessary goal when aiming to organize a sustainable congenital heart program (23).

Although the development of a successful interventional cardiology program depends on the availability of a specialized catheterization laboratory, often times in our experience we choose to team up with available adult catheterization labs, which happen to be ubiquitous in low- and middle-income countries.

Thus, the key issue would be the choice of a person suitable, after proper training, for the role of pediatric interventional cardiologist. When a trained local interventional cardiologist is not available, then the search for an appropriate candidate is conducted among local non-invasive pediatric cardiologists or adult interventional cardiologists. The advantage of the former is a better understanding of the physiology of the heart defects and the familiarity with echo, while the latter have the best technical skills in working with catheterization equipment.

Although catheterization laboratory practice may differ in different countries, nevertheless we would like to point out some common features, typical for almost all catheterization laboratories we have worked with:

1. Significant limitations exist in supplies availability.
  A visiting interventional cardiologist should realize that the majority of diagnostic procedures will have to be performed using a minimal set of diagnostic catheters and guidewires. Berman catheters or wedge balloon catheters are quite rare, as well as delicate equipment used in small children (e.g., 4 French introducers and catheters).
  As most cardiac catheterization materials are disposable, a steady supply chain must be established to provide uninterrupted services. A cost-benefit analysis of certain catheterization procedures has to be weighed against the ready availability of re-sterilizable surgical supplies. For example, a simple surgical PDA ligation may be more cost-effective than device closure in the cardiac catheterization lab when taking into account the cost of sheaths, catheters, wires, and the actual PDA occluder device. However, a similar cost-benefit analysis for procedures requiring open heart surgery may indicate quite the opposite. For instance, surgical closure of an atrial septal defect would require the additional costs of an oxygenator and tubing set, sutures, temporary pacing wires, temporary pacemaker, chest tubes, additional ventilator supplies and ICU disposables, multiple lab tests and blood bank products. Whereas, an uncomplicated transcatheter device closure of an atrial septal defect can be done safely, with minimal ancillary support, transthoracic echocardiographic guidance, and conscious sedation. Another consideration to be addressed are the societal and cultural implications of surgical scars especially in the female patients. Where applicable and safe, transcatheter repair of some defects may be the procedure of choice (51–53).
2. The absence of an anesthesiologist member in the team with enough experience to work with children.
  It should be taken into account during trip planning phase, because most often you have to recruit staff from the ICU team to work in the catheterization lab, unless specific preparations have been made in advance.
3. The need to re-educate staff in adult catheterization labs to work with children.
  The key strategies are the introduction of more delicate vascular access (use of thinner needles and introducers, puncture under echo-guidance, avoidance of unnecessary arterial access); precise monitoring of hemodilution and blood loss (use of small syringes for flushing, accounting the flush volume); temperature management with obligatory patient warming; careful monitoring of hemodynamic changes (arrhythmias, hypotension) with aggressive management and strict infection control, In other words, implementing strategies that usually do not play a significant role in the coronary catheterization lab. As a rule, the introduction of such philosophy takes a significant amount of time, depending on trips frequency and volume of cases.
4. Challenges with hemodynamic evaluation.
  In many catheterization labs in LMIC the absence of a hemodynamic module or its malfunction is common. In these cases, any hemodynamic studies, such as pulmonary hypertension study, become quite problematic. In several cases, we managed to get out of a difficult situation by using an ICU patient's monitor with the possibility to freeze the invasive pressure curve and measure a pressure at a certain point of the curve (e.g., end-diastolic pressure of the systemic ventricle).
5. A bias against routine hemodynamics assessment.
  The tendency for most interventional cardiologists working at sites we have visited, tends to be biased toward performing procedures (e.g., device placement) rather than doing methodological diagnostic catheterizations (54). It is not uncommon to receive Cath reports, performed by local pediatric interventional cardiologists in the LMIC we visit that have incomplete data. This bias although not limited to developing programs, it is widespread among local practitioners and a sensible education process must be implemented early on to change the culture of the site.

In conclusion, the development of the pediatric interventional cardiology program as part of the whole congenital program is depending on maintaining the focus on the three main training strategies to be developed simultaneously:

1. Basic diagnostic and hemodynamic studies, which, combined with perioperative interventions, could provide a basic support for the surgical program. It usually takes about 1 year to develop depending on trips frequency and number of procedures performed on each trip.
2. Neonatal catheterizations and interventions for critical heart defects (55). In our opinion, this is the key strategy for the development of a sustainable congenital program. Firstly, because the learning curve for neonatal interventions for critical defects is significantly shorter than that for emergent neonatal surgery. Secondly, such neonatal interventions as balloon atrial septostomy, aortic or pulmonary valvuloplasty, and in some special patients stenting of PDA or RVOT, which cover the majority of critical heart defects, allowing for the stabilization of the neonate, providing a bridge to correction. The successful development of neonatal interventions will open, in a relatively short period of time (usually 1–2 years depending on frequency and volume), the possibility to provide an emergency services for the most vulnerable category of patients in the covered region. In our experience, such approach significantly increases the credibility of the program by pediatricians and neonatologists in the region, facilitating the organization of a country wide referral network for critical defects, contributing to the development of the program as a whole.
3. Interventions that include balloon valvuloplasty, implantation of stents and devices may, in time, amount to a significant group of patients. Such “curative interventions” could provide an additional motivation for local interventional cardiologists, and eventually bring extra media, financial or political benefits for the congenital program. Unfortunately, devices remain underutilized in LMIC due to high cost. In many countries, device implantation is still a more expensive intervention when compared to open-heart surgery (56).

### Anesthesia Services

At every site we visit, we aim to perform cardiac anesthesia with similar standards as we are used to practice in our countries of origin. In order to do so, there are a few hazards that must be accounted for as we set up.

#### Medication Error

Local drugs are often labeled in languages our anesthesiologists are not familiar with. The packaging dose may differ from the one we are familiar at our home institution. Brand names may or may not be similar. Standardization of effective drug concentration may differ from what it is printed on the label, such as it is the case with some generic drugs produced in laboratories with lower or absent quality control (57). These issues have been particularly noticeable when using Heparin/Protamine from different manufacturers.

Time has to be taken to understand these differences, to draw the drugs to be used in labeled syringes with enough anticipation and, when available, take advantage of the invaluable help a local anesthesiologist or anesthesia technician fluent in both languages may provide, as to avoid potentially lethal iatrogenic mistakes.

#### Blood Loss/Coagulopathy/Blood Products

Hospital blood banks, while always available (a litmus test during our vetting process), tend to be less sophisticated than those in developed countries. This is particularly evident when confronted with the limited offer of fractioned blood products (e.g., Cryoprecipitate and platelets). The theoretical advantage of working in resource limited countries is that whole fresh blood from a family member is almost always readily available, limiting potential cross-reactions while offering young red cells, platelets and factors with a lower Potassium concentration than old blood bank bags (a deadly issue when dealing with neonatal/small infant surgery), at a relatively low cost (58).

Early diagnosis and consequent management of post-operative coagulopathy is not simple unless the proper equipment is available. Unless a specific defect on the coagulation cascade is identified through the timely use of thromboelastography (TEG) or Rotational thromboelastometry (ROTEM), it will be unlikely that a true coagulopathy could be treated before depleting the blood bank reserves and spending hours in the operating room, with the known deleterious effects of hypothermia and multiple transfusions (59). In our experience, as well as others (a) we have found that, when available, the use of HepCon coagulation monitor does improve the monitoring of Heparin/Protamine dosage, to a better degree of accuracy than Activated Clotting Time (ACT Test) while significantly lowering the need for unnecessary transfusions and time in the operating theater (60).

As it is the case in more affluent societies, there is no substitute for good post-operative surgical hemostasis and regulated patient temperature control.

#### Temperature Control

A critical part of open-heart surgery is the ability to control patient body temperature throughout the surgical event and in the early post-operative period. This is especially important in neonates due to their own limitations to self-regulation.

A normal temperature may be easier to achieve on patients undergoing open heart surgery with cardiopulmonary bypass but in many sites less than ideal operating room temperature control may induce early hypothermia and in rare occasions hyperthermia. The lack of external means of temperature control (warm air flow, cooling/warming blankets, etc.) should be a significant concern in the anesthesiologist mind as surgical planning takes place.

#### Time-Out Before Procedures

As part of our daily practice, but most importantly, part of our education process, we have implemented a Time-Out routine in every country we operate regardless of local custom (61).

The process is carried out by our anesthesiologists after draping of the patient but before skin incision. It includes name, age, diagnosis, planned intervention, need for synthetic materials (shunt, patch), planned cannulation, aimed temperature, dose of cardioplegia, planned inotropic support, use of modified-ultrafiltration, and need for residual shunts measurement at the end of the case as well as blood availability and use of perioperative antibiotics.

We have achieved, over the last 4 years, a 95% compliance to the Time-Out routine, independently of primary surgeon (our staff member or a local one).

#### Modified Ultrafiltration (MUF)

We use MUF in all patients under 10 kg and some patients under 15 kg (surgeon's choice) as part of a larger fluid management strategy that is initiated in the operating room and continues in the intensive care unit. The aim of this strategy is to avoid unnecessary fluid overload and to promote early extubation since a fast-track pathway is essential when operating in environments with a limited number of ICU beds that do not allow for prolonged post-operative ventilator support. The extension of this tight fluid management strategy in the ICU includes the early use of a peritoneal dialysis catheter on post-operative day one when urine output is less than satisfactory.

#### Limitations to Post-operative Evaluation

Poor cardiac function after aortic cross-clamp, residual air in left sided cavities and residual shunts play a significant role in the postoperative morbidity and mortality of these populations. The inability to rule out these issues by performing trans-esophageal (TEE) ultrasound, either due to absence of trans-esophageal probe (a common occurrence in our experience) or lack of a second ultrasound machine for exclusive use in the operating theater undoubtedly play a significant role in the higher morbidity/mortality.

This issue may be palliated by epicardial echocardiogram (when a machine is available) using a sterile sleeve and the regular trans-thoracic probe. In the absence of TEE or Epicardial echo, as it is the case in most of our surgical sites, we have tried to implement an alternative substitute by performing a residual shunt calculation by direct measurement (SVC and PA) on 50% or less FIO2. While this procedure prolongs the anesthesia time by 15 min or so, it may avoid an unplanned trip to the operating theater for reoperation of residual intra-cardiac shunting and a protracted postoperative ICU stay. Likewise, while still in the operating room often we use direct measurements of hemodynamics (needle and sterile pressure tubing) to measure blood pressures in different cavities as well as residual gradients.

### Perfusion Services

Currently the state of formal perfusion education in the LMICs we service is virtually non-existent. Since the ability to attend perfusion school is unfeasible, many LMIC perfusionists learn via an apprentice model as it was the case in the USA in the 1960s (61) Although some sites already have experience with perfusion (particularly adults) almost all sites require long term intensive perfusion assistance. This enables the techniques, unique to pediatric perfusion, to be fully and safely implemented. Utilizing experienced pediatric perfusionists certified in Western countries who may have clinical experience based on hundreds of perfused cases combined with a strong base knowledge to draw from, enables a rich educational experience for the local perfusionist.

Perfusion circuitry is single use medical equipment, so the availability of this equipment varies by country. Operating in many different locales, we can appreciate the need to perform perfusion techniques differently albeit safely. To maintain a united front in our varied practices' we adhere to The American Society of Extracorporeal Technology Standards and Guidelines for Perfusion Practice which have allowed us to enact meaningful change, including pre-bypass checklists and intra operatively charting of perfusion parameters at our sites (62).

Pediatric perfusion has become a niche practice and now even offers a fellowship credential (63). As the practice of pediatric perfusion grows every day, more literature becomes available inclusive of technique articles that we have published in peer reviewed, open access journals (64, 65). We continue to explore new means to conduct pediatric perfusion while maintaining optimal outcomes during our goal of producing expert perfusion clinicians which in turn can train the next local generation.

### Biomedical Engineering

Working in LMIC is challenging and one area where it can be especially difficult is equipment. The site questionnaire will give you some indication of the equipment present but until the site visit you cannot be sure if the equipment listed is actually functional and has all components needed. We routinely solicit donations of equipment from hospitals and clinics when they upgrade. Donated used equipment from the United States or Canada to be used abroad must be check for capability of dual power input (110v/220v) otherwise a voltage converter or transformer should also be included in the donation. The need for a biomedical engineer on each trip is paramount to the success of a surgical trip, particularly when visiting sites with old, refurbished or poorly maintained equipment. The individual should have a broad range of experience as repair can be required on bypass units, heater/coolers, electro-cautery units, surgical overhead and fiber-optic headlights as well as a variety of anesthesia machines. The ventilators, monitors, infusion and syringe pumps in the ICU also can come from a variety of manufacturers. Also, we utilize our biomedical engineer to assess and familiarize himself with the hospitals back-up generator and oxygen sources, whether central or tank supply.

### Operating Room Services

Work in the cardiac operating room department involves multidisciplinary teamwork including anesthesia, perfusion, OR nurses and scrub technicians. The operating room (OR) team of NCA works in close collaboration with the local OR team to provide safe and successful surgical outcome. Prior the first inaugural trip NCA surgeon, perioperative nurse and biomedical engineer communicate with local staff to ensure that all essential medications, disposables, and equipment are available locally. In general, host hospitals are responsible to provide surgical instruments, medicines and supplies necessary to provide open heart surgery. Items not obtainable locally are provided by NCA with the help of donations from medical companies and private donations and shipped to the hospital. In many instances NCA team is responsible to provide instrument sets for surgeries because new centers are not equipped with delicate and specific pediatric tools. Many centers lack emergency opening set which is designated for emergency chest opening in the ICU. The centers that commit to a program building often receive request from NCA surgeon to secure at least two full surgical sets and one emergency set. Other essential equipment that are usually lacking at new sites are surgical headlight, reliable blood gas machine, oscillating saw and infant/pediatric defibrillator paddles. NCA team would bring all this equipment per trip needs and eventually as the program grows the host hospital secures those items locally.

Procurement of medical supplies for surgical trips is extensive and ongoing. Many countries require a full support from NCA to provide disposable materials for surgeries. Thanks to donations from medical companies, non -profit charities and hospitals from the United States we are able to meet the challenges and provide necessary supplies. Many donated items are close to expiration date or expired and some countries are willing to accept those items while other require a minimum of 1 year of a shelf life for the same items. Expired supplies are repackaged and re sterilized in host country according to the type of material in autoclave, ethylene oxide or formaldehyde sterilization process for the heat sensitive items.

One of the main reasons why NCA is traveling to low income countries is to provide education in pediatric cardiac surgery and to bring the latest standards and practices in pediatric cardiac care to local physicians and nurses.

In the countries where we travel, we have experienced the shortage of cardiac anesthesiologists, OR nurses and pediatric perfusionists. Due to the shortage of personnel, many hospitals would offer us the group of doctors and nurses to teach but only few of them would accept to continue with education. The problem that we face in low income countries is that these professionals are doing two jobs in order to meet living needs and they simple cannot afford to spend more time in the hospital. In order to be successful, we need to be aware of the culture and sensitive to the needs of the host. Therefore, our educational approach must meet the needs that would go side by side with their respective beliefs. The education is conducted in English language. Most physicians speak English language while the main challenges are with non-English-speaking nurses. In that case we would ask physician to translate, provide on site medical translator and translate educational materials in a local language. Most commonly we work side by side with a local nurse and doctor and monitor their work closely.

Each center is required to perform a time out before the procedure begins to ensure that the entire surgical team is familiar with the patient, procedure and that patient is ready for the surgery (66, 67).

The role of NCA operating room staff is not only to teach about particular surgery but also to provide education and additional information in all other aspects of the perioperative department from cleaning, disinfection and sterilization to keeping up with correct temperature, relative humidity and air currents to prevent airborne transmission of infectious particles (68).

### Providing Service in Conflict Zones

For the most part, global conflicts have been limited to LMIC since the end of World War ll. However, terrorists' attacks have occurred in several European countries in addition to the United States as well as LMIC. The emergence of Al Qaeda and ISIS in the Middle East, North Africa and Central Asia over the last 18 years has created armed conflict zones in LMIC where previously there were none. The world has become a more dangerous place over the last two decades and nowhere is totally safe (69, 70). Since the most underserved regions of the world for children with heart disease are LMIC, the chances of being in or near a conflict zone are considerable (71). Children located in or near conflict zones deserve our assistance just as much as children anywhere else. Security and safety for the team is paramount and an objective assessment should be made by experts coupled with trusted individuals on-site if you are entertaining working in a conflict zone (72). Additionally, the Foreign Ministries or State Departments in the countries of origin of the visiting team can also provide you with security assessments/warnings. Those doing humanitarian work in a conflict zone should notify their Embassy at the place of deployment, with arrival and departure dates and a local telephone number to contact you should a change in events warrant early departure/evacuation. Our teams have been evacuated twice in our 27-year history, from Belgrade after the NATO bombing started in 1999 and in Benghazi in 2014 when the civil war erupted in that city. On both occasions we were evacuated by the Ministry of Interior and no team member suffered any physical or significant psychological injury. We have been in conflict zones when fighting started nearby but did not require evacuation; West Bank with the second Intifada, Pakistan when Al Qaeda attacked the Pakistan Army's General Headquarters, Iraq with invasion of ISIS and Tripoli when a militia attempted to overthrow the government. When working in such areas it is critical to have daily security updates in order to make decisions insuring the safety of the team.

### Benchmarks and Indicators of Improvement

We are pleased and rewarded when we provide or assist in successful interventions that improve the life of a child providing them with the opportunity of a future. Such altruistic action benefits the child, removes psychological and financial burdens from the family and is seen by the local staff as a reward for their hard work. Each intervention must also serve as an educational event in order to advance the experience of the local healthcare providers. The goals set for the program prior to beginning must be focused on with every trip. We establish benchmarks for achievement and will frequently focus on a particular type of defect during a trip, collecting a few cases so that we can allow the local team to assume primary responsibility for all phases of care. Careful mentorship and active assistance are needed so that success is achieved, and confidence is instilled in the local team. We are responsible for the safety of the child first but maintaining the moral of the local team is essential for their progress and development of confidence.

Earlier we mentioned the need for a local database and one of the benefits is that both the visiting team and local team can see the interval progress made when the visiting team is absent. We cannot overstate the need for interval advancement assessment by both participants in this collaborative effort to improve pediatric cardiac care. We also recommend that each site participate in the International Quality Improvement Collaborative in Pediatric Cardiac Disease (IQIC) (73). A review of interval work formally or informally should be done at the beginning of each trip. We have found that this provides us, the teachers, with the information needed to decide whether to take the next step or continue to work on the previous defect until satisfactory results are obtained. Our approach is to review interval data for volume of work done, complexity of defects receiving intervention, mortality, morbidity along with ICU and hospital length of stay. A comprehensive annual review with comparison to previous year(s) should be performed and statistically analyzed if possible. The IQIC provides reports to each site as well so that the site can compare themselves to similar programs (74).

The registry of patients evaluated allows the local team to assess the impact of their program by enabling them to determine if the volume of patients being evaluated is increasing. Additionally, the registry can provide the local team information regarding prevalence of defects within their referral area. Moreover, by maintaining and reviewing the registry the local team can assure that patients are seen and followed in a timely fashion thereby possibly preventing some of the complications of palliative and untreated CHD.

### Quality Improvement/Assessment

A review of the work performed on the visit to highlight improved areas as well as discussing issues that remain organizationally is important and we combine this with a mortality and morbidity discussion. One must be attuned to the cultural issues when conducting this review/de-briefing and avoid personalizing failures. Catastrophic failures resulting in deaths should be addressed immediately so if systems failure is the issue it can be corrected to prevent future failures. If the catastrophe is the result of an individual's mis-adventure a direct discussion privately with the involved individual(s) should be conducted (75). Transparency and honesty are needed if personnel and program growth is to continue. The QI/A meeting should be a regular occurrence on a monthly basis conducted by the local team.

System failures may be organizational and more easily solved than infrastructural ones. We must be aware that expensive alterations in infrastructure may require significant time to correct and short-term alternative options may be needed. We have partnered with sponsors both abroad and locally in some cases to provide expensive infra-structure improvements. When then issue is exclusively limited to equipment failure, then donation of refurbished equipment is something that is much easier to solve, keeping in mind differences in electrical current types and furnishing the appropriate transformers along with the devices.

### Ethical Considerations

Ethical issues are to some degree dependent upon the location and culture of the country you are assisting (76). Some cultural beliefs espouse a policy of no withdrawal of life support even in patients declared brain dead. Others forbid extended visitation in the ICU and still others do not want to “*waste resources*” on children with chromosomal abnormalities. Whereas, you may be the expert in congenital heart disease you may be ignorant of ethical differences depending on the country. It is always best to defer to the hosts in delicate issues unless they impact patient care. One example would be a site which does not want to utilize the resources for open repair cases in patients with Trisomy 21 but will reluctantly agree to a palliative procedure (77). You may want to avoid a controversial situation which could potentially result in an argument and disrupt the relationships you are trying to develop, while trying to explain the decreased burden on the health care system if such patient receives a complete repair (78). Navigating situations like these can be difficult, but we have successfully removed the stigmata of “Downs Syndrome operations” in every country that has initially resisted. Religious and ethnic differences are also excuses used to deny operations in some countries and one must work hard to overcome this discrimination against a child. The differences in gender approach can result in very uncomfortable to outright disastrous situations if one is not cognizant of cultural differences (79). When one is dealing with someone of a different gender it is always helpful to allow the hosts to lead the approach whether it is with a greeting, performance of a physical or diagnostic exam. All trips eventually come to an end and this can be the most stressful and ethically challenging time of the visit. Once parents realize that your visit is nearing an end, they may become frantic if their child is not on the operative list. Moreover, they will at times hide critical information from the team regarding recent infections. Also, it is not uncommon that some child with complex defect appears at the end of the trip and both the local team and parents are expecting an operation (80). We routinely provide an ICU stay over team in the initial stages of our programs, but even in this situation we prefer to decrease the complexity of the cases performed so that the ICU acuity is reduced, and we are confident that the stay over team can depart without anyone in the ICU. Performing a complex repair on the last days of a visit that will require ICU care beyond the time limits of the stay over team is an ethical challenge and has no simple answer.

## Summary

Pediatric cardiac care is deficient in most of the world and as a result hundreds of thousands of children with congenital and rheumatic heart disease die annually. The majority of population growth is in LMIC, the very regions where deficiencies in pediatric cardiac care exist. Bringing children out of their countries for surgery in more developed healthcare systems results at best in a few hundred receiving life-saving care at a prohibit cost for the family or the sponsoring government or institution. The only long-term solution is to build local capacity. We have described our models which have all led to the development of successful programs, but we have failures as well. The choice of the site for development is critical, but cannot always predict the imponderables of politics, conflict, financial collapse, or personnel failures.

## Author Contributions

All authors listed have made a substantial, direct and intellectual contribution to the work, and approved it for publication.

### Conflict of Interest Statement

The authors declare that the research was conducted in the absence of any commercial or financial relationships that could be construed as a potential conflict of interest.

## References

[1] Neirotti R. Paediatric cardiac surgery in less privileged parts of the world. Cardiol Young. (2004) 14:341–6. 10.1017/S1047951104003191 15680035

[2] Tchervenkov CI Jacobs JP Bernier PL Stellin G Kurosawa H Mavroudis C. The improvement of care for pediatric and congenital cardiac disease across the World: a challenge for the World Society for Pediatric and Congenital Heart Surgery. Cardiol Young. (2008) 18 (Suppl. 2):63–9. 10.1017/S1047951108002801 19063776

[3] Bernier PL Stefanescu A Samoukovic G Tchervenkov CI. Tchervenkov. The challenge of congenital heart disease worldwide: epidemiologic and demographic facts. Semin Thorac Cardiovasc Surg Pediatr Card Surg Ann. (2010) 13:26–34. 10.1053/j.pcsu.2010.02.005 20307858

[4] World Heart Federation RHD Affects the World's Poorest, Most Vulnerable Populations. (2018). Available online at: https://www.world-heart-federation.org/programmes/rheumatic-heart-disease (accessed July 15, 2018).

[5] Gross RE Hubbard JP Surgical ligation of a patent ductus arteriosus: report of first successful case. JAMA. (1939) 112:729–31. 10.1001/jama.1939.02800080049011 6363741

[6] Crafoord C Nylin G Congenital coarctation of the aorta and its surgical treatment. J Thorac Surg. (1945) 14:347.

[7] Blaylock A Tassig HB Surgical treatment of malformations of the heart: in which there is pulmonary stenosis or pulmonary atresia. JAMA. (1945) 128:189–202. 10.1001/jama.1945.02860200029009 6368878

[8] Warden HE Cohen M Read RC Lillehei CW. Controlled cross circulation for open intracardiac surgery: physiologic studies and results of creation and closure of ventricular septal defects. J Thorac Surg. (1954) 28:331–41; discussion, 341–3. 13192880

[9] Lillehei CW Cohen M Warden HE Varco RL. The direct-vision intracardiac correction of congenital anomalies by controlled cross circulation; results in thirty-two patients with ventricular septal defects, tetralogy of Fallot, and atrioventricularis communis defects. Surgery. (1955) 38:11–29. 14396676

[10] Kirklin JW Dushane JW Patrick RT Donald DE Hetzel PS Harshbarger HG. Intracardiac surgery with the aid of a mechanical pump-oxygenator system (gibbon type): report of eight cases. Proc Staff Meet Mayo Clin. (1955) 30:201–6. 14371757

[11] Nguyen N Leon-Wyss J Iyer KS Pezzella AT. Paediatric cardiac surgery in low-income and middle-income countries: a continuing challenge. Arch Dis Child. (2015) 100:1156–9. 10.1136/archdischild-2015-308173 26359507

[12] Replogle RL International Community of Cardiac Surgeons. Ann Thorac Surg. (1996) 62:635 10.1016/S0003-4975(96)00560-7

[13] McGrath LB. Establishing a pediatric cardiac surgical unit in the Commonwealth of Independent States (formerly the Soviet Union). J Thorac Cardiovasc Surg. (1992) 104:1758–9. 1453748

[14] Lansing LM. Heart surgery in underdeveloped countries. Success in Panama and Romania. Ann Thorac Surg. (1993) 56:1439–40. 10.1016/0003-4975(93)90718-W 8267463

[15] Cohen AJ Tamir A Houri S Abegaz B Gilad E Omohkdion S. Save a child's heart: we can and we should. Ann Thorac Surg. (2001) 71:462–8. 10.1016/S0003-4975(00)02243-8 11235690

[16] Novick WM Stidham GL Karl TR Guillory KL Ivanćan V Malcić I. Are we improving after 10 years of humanitarian paediatric cardiac assistance? Cardiol Young. (2005) 15:379–84. 10.1017/S1047951105000806 16014185

[17] Novick WM Stidham GL Karl TR Arnold R Anić D Rao SO. Paediatric cardiac assistance in developing and transitional countries: the impact of a fourteen year effort. Cardiol Young. (2008) 18:316–23. 10.1017/S1047951108002175 18405428

[18] Pezzella AT. International aspects of cardiac surgery. Ann Thorac Surg. (1998) 65:903–4. 9564897 10.1016/s0003-4975(98)00091-5

[19] Neirotti RA. Cardiac surgery: the infinite quest. Rev Bras Cir Cardiovasc. (2012) 27:614–20. 10.5935/1678-9741.20120104 23515735

[20] Dearani JA Neirotti R Kohnke EJ Sinha KK Cabalka AK Barnes RD. Improving pediatric cardiac surgical care in developing countries: matching resources to needs. Semin Thorac Cardiovasc Surg Pediatr Card Surg Annu. (2010) 13:35–43. 10.1053/j.pcsu.2010.02.001 20307859

[21] Dearani JA Jacobs JP Morton Bolman R Swain JD Vricella LA Weinstein S. Humanitarian outreach in cardiothoracic surgery: from setup to sustainability. Ann Thorac Surg. (2016) 102:1004–11. 10.1016/j.athoracsur.2016.03.062 27319988

[22] Neirotti RA. Barriers to development pushing the boundries. Rev Bras Cir Cardiovasc. (2015) 30:104–13. 10.5935/1678-9741.20150007 25859874 PMC4389531

[23] Polivenok IV Molloy FJ Gilbert CL Danton M Dodge-Khatami A Rao SO. Results of international assistance for a paediatric heart surgery programme in a single Ukrainian centre. Cardiol Young. (2019) 29:363–8. 10.1017/S1047951118002457 30813981

[24] Swain JD Sinnott C Breakey S Charles RH Mody G Nyirimanzi N. Ten-year clinical experience of humanitarian cardiothoracic surgery in Rwanda: building a platform for ultimate sustainability in a resource-limited setting. JTCVS. 155:2541–50. 10.1016/j.jtcvs.2017.11.106 29499865

[25] Young JN Everett J Simsic JM Taggart NW Litwin SB Lusin N. A stepwise model for delivering medical humanitarian aid requiring complex interventions. JYCVS. (2014) 148:2480–9. 10.1016/j.jtcvs.2014.07.067 25263713

[26] Welke KF O'Brien SM Peterson ED Ungerleider RM Jacobs ML Jacobs JP. The complex relationship between pediatric cardiac surgical case volumes and mortality rates in a national clinical database. J Thorac Cardiovasc Surg. (2009) 137:1133–40. 10.1016/j.jtcvs.2008.12.012 19379979

[27] Welke KF Diggs BS Karamlou T Ungerleider RM. The relationship between hospital surgical case volumes and mortality rates in pediatric cardiac surgery: a national sample, 1988–2005. Ann Thorac Surg. (2008) 86:889–96. 10.1016/j.athoracsur.2008.04.077 18721578

[28] Shah AA Aftab M Tchantchaleishvili VT LaPar DJ Stephens EH Walters DM. Characterizing the operative experience of cardiac surgical trainees: what are residents really doing in the operating room? Ann Thorac Surg. (2016) 101:2341–9. 10.1016/j.athoracsur.2015.12.069 27021035

[29] Vaporciyan AA Yang SA Baker CJ Fann JI Verrier ED. Cardiothoracic surgery residency training: past, present, and future. JTCVS. (2013) 146:759–67. 10.1016/j.jtcvs.2013.06.004 23870155

[30] Gabler NB Ratcliffe SJ Wagner J Asch DA Rubenfeld GD Angus DC Mortality among patients admitted to strained intensive care units. Am J Respir Crit Care Med. (2013) 108:800–6. 10.1164/rccm.201304-0622OC PMC3826272 23992449

[31] Montgomery VL. Effect of fatigue, workload, and environment on patient safety in the pediatric intensive care unit. Pediatr Crit Care Med. (2007) 8 (Suppl. 1):S11–6. 10.1097/01.PCC.0000257735.49562.8F 17496827

[32] Fortenberry JD Making the pediatric intensive care unit a safe haven. Pediatr Crit Care Med. (2007) 8 (Suppl. 1):S1–2. 10.1097/01.PCC.0000257734.84591.1F

[33] Larrazabal LA Jenkins KJ Gauvreau K Vida VL Benavidez OJ Gaitán GA. Improvement in congenital heart surgery in a developing country the guatemalan experience. Circulation. (2007) 116:1882–7. 10.1161/CIRCULATIONAHA.107.695403 17965404

[34] Leon-Wyss JR Veshti A Veras O Gaitán GA O'Connell M Mack RA Gaitán pediatric cardiac surgery: a challenge and outcome analysis of the guatemala effort. Semin Thorac Cardiovasc Surg Pediatr Card Surg Ann. (2009) 12:8–11. 10.1053/j.pcsu.2009.01.003 19349009

[35] Maher KO Chang AC Shin A Hunt J Wong HR. Innovation in pediatric cardiac intensive care: an exponential convergence toward transformation of care. World J Pediatr Congenit Heart Surg. (2015) 6:588–96. 10.1177/2150135115606087 26467873

[36] Checchia PA Laussen PC Macrae D Bohn D Chang AC Wessel DL. Pediatric cardiac intensive care: a transition to maturity. Pediatr Crit Care Med. (2016) 17 (8 Suppl. 1):S110–1. 10.1097/PCC.0000000000000824 27490587

[37] Stocker M Pilgrim SB Burmester M Allen ML Gijselaers WH. Interprofessional team management in pediatric critical care: some challenges and possible solutions. J Multidiscip Healthc. (2016) 9:47–58. 10.2147/JMDH.S76773 26955279 PMC4772711

[38] Fenton KN Castillo SH Claro CD Novick WM. Teamwork and program organization in developing countries. World J Pediatr Cong Heart Surgery. (2011) 2 219–24. 10.1177/2150135110395334 23804975

[39] Almandel A Younis H Gilbert C Marr S Bowtell K Forsberg B Pediatric cardiac services in Iraq: current status and future plans. J. Cardiothor Surg. (2013) 8 (Suppl. 1):O303 10.1186/1749-8090-8-S1-O303

[40] Lou X Lee R Feins RH Enter D Hicks GL Jr Verrier ED. Training less-experienced faculty improves reliability of skills assessment in cardiac surgery. J Thorac Cardiovasc Surg. (2014) 148:2491–6. 10.1016/j.jtcvs.2014.09.017 25308119

[41] Kogon B Karamlou T Baumgartner W Merrill W Backer C. Congenital cardiac surgery fellowship training: a status update. J Thorac Cardiovasc Surg. (2016) 151:1488–95. 10.1016/j.jtcvs.2016.02.039 27002229

[42] Babb DA Salerno TA. Setting the standard: the necessity of internationalizing residency training programs. J Thorac Cardiovasc Surg. (2018) 157:2547–8. 10.1016/j.jtcvs.2018.10.118 30503735

[43] Starnes VA Sullivan ME. The American board of thoracic surgery congenital fellowship: have we lost our international heritage? Semin Thorac Cardiovasc Surg Pediatr Card Surg Annu. (2017) 20:77–8. 10.1053/j.pcsu.2016.09.006 28007070

[44] Stawicki SP Nwomeh BC Peck GL Sifri ZC Garg M Sakran JV. Training and accrediting international surgeons. BJS. (2019) 106:e27–33. 10.1002/bjs.11041 30620074

[45] Novick W Molloy F Cardarelli M Gilbert C Pediatric Cardiac Development Assistance in Iraq: 2012–2016. Cardiol Young. (2017) 27 (Suppl. 4):S227.

[46] Novick W Amer W Cardarelli M Rodriguez H Pediatric cardiac assistance and development in Eastern Libya. Cardiol Young. (2017) 27 (Suppl. 4):S228.

[47] Busse H Aboneh EA Tefera G. Learning from developing countries in strengthening health systems: an evaluation of personal and professional impact among global health volunteers at Addis Ababa University's Tikur Anbessa Specialized Hospital (Ethiopia). Global Health. (2014) 10:64. 10.1186/s12992-014-0064-x 25190076 PMC4172777

[48] McCrary NE Mazur JM Conceptualizing a narrative simulation to promote dialogic reflection: using a multiple outcome design to engage teacher mentors. Edu Technol Res Dev. (2010) 58:325–42. 10.1007/s11423-008-9100-y

[49] Satish U Streufert S. Value of a cognitive simulation in medicine: towards optimizing decision making performance of healthcare personnel. Qual Saf Health Care. (2002) 11:163–7. 10.1136/qhc.11.2.163 12448810 PMC1743599

[50] Lapkin S Levett-Jones T Bellchambers H Fernandez R Effectiveness of patient simulation manikins in teaching clinical reasoning skills to undergraduate nursing students: a systematic review. Clin Simulat Nurs. (2010) 6:207–22. 10.1016/j.ecns.2010.05.005 27820553

[51] Law MA Grifka RG Mullins CE Nihil MR. Atrial septostomy improves survival in select patients with pulmonary hypertension. Am Heart J. (2007) 153:779–84. 10.1016/j.ahj.2007.02.019 17452153

[52] Almanla A Charafeddine F Abutaqa M Mustafa H Tabbakh A Hussein HB. Transcatheter closure of atrial septal defects: comparable experience and outcomes between developing and developed countries. Pediatr Cardiol. (2019) 40:610–5. 10.1007/s00246-018-2034-1 30607441

[53] Askari B Soraya H Ayremlu N Golmohammadi M. Short-term outcomes after surgical versus trans catheter closure of atrial septal defects: a study from Iran. Egypt Heart J. (2018) 70:249–53. 10.1016/j.ehj.2018.09.003 30591738 PMC6303347

[54] Alwi M. Stenting the ductus arteriosus: case selection, technique and possible complications. Ann Pediatr Cardiol. (2008) 1:38–45. 10.4103/0974-2069.41054 20300236 PMC2840730

[55] Alwi M Choo KK Latiff HA Kandavello G Samion H Mulyadi MD. Initial results and medium-term follow-up of stent implantation of patent ductus arteriosus in duct-dependent pulmonary circulation. J Am Coll Cardiol. (2004) 44:438–45. 10.1016/j.jacc.2004.03.066 15261945

[56] Glatz AC Petit CJ Goldstein BH Kelleman MS McCracken CE McDonnell A. Comparison between patent ductus arteriosus stent and modified blalock-taussig shunt as palliation for infants with ductal-dependent pulmonary blood flow. Circulation. (2017) 137:589–601. 10.1161/CIRCULATIONAHA.117.029987 29042354

[57] Frenkiel O Overton I O'Connor K Bad Medicine: The Counterfeit Drug Industry is Huge and the Profits are Vast. Some are Harmless but Others are Lethal. Available online at: http://news.bbc.co.uk/2/hi/programmes/this_world/4656623.stm (accessed November 9, 2018).

[58] Manno CS Hedberg KW Kim HC Bunin GR Nicolson S Jobes D Norwood. Blood. (1991) 77:930–6. 1995100

[59] Whiting D DiNardo JA. teg and rotem: technology and clinical applications. Am J Hematol. (2014) 89:228–32. 10.1002/ajh.23599 24123050

[60] Despotis GJ Joist JH Hogue CW Jr Alsoufiev A Kater K Goodnough LT. The impact of heparin concentration and activated clotting time monitoring on blood conservation: a prospective, randomized evaluation in patients undergoing cardiac operation. J Thorac Cardiovasc Surg. (1995) 110:46–54. 10.1016/S0022-5223(05)80008-X 7609568

[61] White MC Randall K Capo-Chichi NFE Sodogas F Quenum S Wright K. Implementation and evaluation of nationwide scale-up of the Surgical Safety Checklist. BJS. (2019) 106:e91–102. 10.1002/bjs.11034 30620076 PMC6519364

[62] Plunkett PF. Perfusion education in the USA. Perfusion. (1997) 12:233–41. 10.1177/026765919701200405 9234395

[63] AmSECT The American Society of ExtraCorporeal Technology Standards and Guidelines for Perfusion Practice. (2019). Available at: http://www.amsect.org/p/cm/ld/fid=1617 (accessed October 25, 2018).

[64] AmSECT AmSECT Fellow of Pediatric Perfusion. (2019). Available online at http://www.amsect.org/page/pediatric-fellow-requirements

[65] Forsberg BC Novick WM. A simplified approach to pediatric modified ultrafiltration: a novel circuit design. J Extra Corpor Technol. (2013) 45:259–61. 24649576 PMC4557501

[66] Croti UA Jenkins KJ Braile DM. Checklist in pediatric cardiac surgery in Brazil: an useful and necessary adaptation of the Quality Improvement Collaborative International Congenital Heart Surgery in Developing Countries. Rev Bras Cir Cardiovasc. (2011) 26:511–5. 10.5935/1678-9741.20110034 22086596

[67] Congenital Checklist Congenital Heart Surgery Checklist. (2018). Available online at: https://www.sts.org/sites/default/files/files/PDF/CongenitalChecklist2016.pdf (accessed January 4, 2019).

[68] Hauk L Periop breifing: did you know. AORN J. 2 108:14. 10.1002/aorn.12318

[69] Boyle D Horton H Yorke H Sawer P Westminster 'Terror Attack': Driver Arrested after Car Mows Down Cyclists and Ploughs into Parliament Barrier. Available online at: www.telegraph.co.uk/news/2018/08/14/car-crashes-barrier-outside-parliament-armed-police-surround (accessed September 19, 2018).

[70] Jenkins N A Timeline of Recent Terrorist Attacks in Europe. (2018). Available online at: http://time.com/4607481/europe-terrorism-timeline-berlin-paris-nice-brussels/ (accessed September 18, 2018).

[71] Hoffman JIE. The global burden of congenital heart disease. Cardiovasc J Afr. (2013) 24:141–5. 10.5830/CVJA-2013-028 24217047 PMC3721933

[72] Zakaria T Cornwell S Al Shalchi H For Benghazi Diplomatic Security, U.S. Relied on Small British Firm. Available online at: www.reuters.com/article/us-libya-usa-bluemountain/for-benghazi-diplomatic-security-u-s-relied-on-small-british-firm-idUSBRE89G1TI20121018 (accessed September 19, 2018).

[73] Jenkins KJ Castañeda AR Cherian KM Couser CA Dale EK Gauvreau K. Reducing Mortality and Infections After Congenital Heart Surgery in the Developing World. Pediatrics. (2014) 134:e1422–e1430. 10.1542/peds.2014-0356 25311607

[74] Schidlow DN Jenkins KJ Gauvreau K Croti UA Giang DTC Konda RK. Transposition of the great arteries in the developing world surgery and outcomes. J Am Coll Cardiol. (2017) 69:43–51. 10.1016/j.jacc.2016.10.051 28057249 PMC7144419

[75] Novick WM Fenton KN Chapter: The ethics, power and influence of leadership. In: Firstenberg M Stawicki S, editors. Fundamentals of Leadership for Healthcare Professionals. Hauppauge, NY: Nova Publishing (2018).

[76] Steyn E Edge J. Ethical considerations in global surgery. BJS. (2019) 106:e17–9. 10.1002/bjs.11028 30588613

[77] Fenton Kathleen N. Cardarelli Marcelo Molloy Frank Novick William M.. Ethics in humanitarian efforts: when should resources be allocated to paediatric heart surgery? Cardiol Young. (2019) 29:36–9. 10.1017/S1047951118001713 30334497

[78] Cardarelli M Vaikunth S Mills K DiSessa T Molloy F Sauter E. Cost-effectiveness of humanitarian pediatric cardiac surgery programs in low-and middle-income countries. JAMA Netw Open. (2018) 1:e184707. 10.1001/jamanetworkopen.2018.4707 30646368 PMC6324367

[79] Shrime MG Sleemi A Ravilla TD. Charitable platforms in global surgery: a systematic review of their effectiveness, cost-effectiveness, sustainability, and role training. World J Surg. (2015) 39:10–20. 10.1007/s00268-014-2516-0 24682278 PMC4179995

[80] Kinsley RH. The third Aldo Castaneda lecture: the neglect of neonatal/infant cardiac disease in Africa–continental genocide? World J Pediatr Congenit Heart Surg. (2012) 3:241–3. 10.1177/2150135111425394 23804781

