# Supplementary material for: Pediatric Cardiac Service Development Programs for Low- and Middle-Income Countries in Need of Improving or Initiating Local Services
Source: Front Pediatr. 2019 Sep 20;7:359. doi: 10.3389/fped.2019.00359 (PMC6763596; doi:10.3389/fped.2019.00359)
Supplement: Supplementary file 1 [file Data_Sheet_1.doc]

**APPENDIX 1**

**SITE ASSESSMENT QUESTIONNAIRE:**

The Novick Cardiac Alliance would like to evaluate your institutions ability to support a trip by the Foundation to provide cardiac diagnosis and surgery to children. The questionnaire is generic and you will find questions, which may or may not be appropriate to your specific institution. Please make an effort to answer each question as completely as possible. Questions, which are not applicable, can simply be answered with NA.

**Hospital Infrastructure: General**

1. Is you institution a general hospital or a pediatric hospital or a cardiac institution? How many total beds in your institution?
2. What is the official language spoken in your institution? Does many of the professional staff speak English?
3. If you institution is not a pediatric hospital what pediatric sub-specialties are present in your hospital?
4. What type of electrical power do you use in your hospital? 60 cycle/110-120 volts or 50 cycle/220-240 volts?
5. What is the configuration of the electrical outlet? Standard US or Standard European or other? If other please send photo of electrical outlet.
6. Do you have emergency generators that work if power is interrupted?
7. If the central water supply is interrupted do you have an alternative source for water within the hospital?
8. Is the oxygen source for the operating rooms and the intensive care unit delivered from a central source or by cylinders located in or near these rooms? What is the operating pressure if the oxygen is supplied from a central source? If a central supply, what is the manufacturer and model of the outlet? Please send photo of gas outlets.
9. Do you have a central source for air delivery in the operating rooms and intensive care unit or by cylinders located in or near these rooms? ? What is the operating pressure if the air is supplied from a central source? If a central supply what is the manufacturer and model of the outlet? Please send photo of air outlet.
10. Do you have a central source for suction (vacuum) in the operating rooms and intensive care unit or do individual machines in these areas provide it?

# Hospital infrastructure: Intensive care unit

1. Is the intensive care unit located on the same floor as the operating rooms? If yes, how far from the operating rooms is the intensive care unit?
2. If the intensive care unit is not located on the same floor as the operating rooms please describe how the patients are transferred to the intensive care unit following operation and how far this distance is from the operating rooms. If there is an elevator involved in the transport of patients from the operating room to intensive care unit is this elevator dedicated for that purpose or shared for other purposes?
3. Do you have a separate intensive care unit for patients following heart surgery? If yes, how many beds are in this intensive care unit?
4. If you do not have a separate intensive care unit for heart surgery patients what intensive care unit do you use to recover the heart surgery patients? How many beds in this intensive care unit are available for heart surgery patients?
5. If your hospital is not a pediatric hospital, does it have a separate pediatric intensive care unit? If yes, how many beds does it contain?
6. Is the intensive care unit that is used to recover the heart surgery patient’s air-conditioned?
7. How many sources of oxygen are available at each bed space, one or two or more?
8. How many sources for air are available at each bed space, one or two or more?
9. How many sources for suction are available at each bed space, one or two or more?
10. How many electrical outlets are available at each bed space?
11. Does each bed space have a ventilator? Please give the manufacturer and model.
12. Does each bed space have an invasive pressure monitor capable of monitoring two invasive pressures simultaneously? Does the monitor have pulse oximetry capability? If no pulse oximetry is available on this monitor do you have separate pulse oximetry machines for each bed space? Please give the manufacturer and model.
13. How many infusion pumps for delivery of intravenous infusions are available for each bed space?
14. Do you have syringe pumps available for the delivery of inotropic infusions to children? If yes, how many are available for each bed space?
15. Do you have radiant warmers available to warm children following heart surgery? If yes, how many are available?
16. Do you have defibrillators available in the intensive care unit? If yes, do you have sterile internal paddles also available in the intensive care unit? If yes, do you have pediatric sizes?
17. Do you have temporary pacemaker pulse generators available in the intensive care unit? Please list how many and the manufacturer and model.
18. Do you have a sterile thoracotomy tray in the intensive care unit for emergency chest reopening? Does it contain pediatric size retractors?
19. Does the intensive care unit have the capability of performing laboratory analysis on site, or must all samples go to the central laboratory?

**Personnel:**

1. Who is responsible for the care of patients following heart surgery? Surgeons or anesthesiologists or intensive care unit physicians or cardiologists?
2. Does the physician caring for heart surgery patients following surgery have any experience caring for children following operation?
3. Have any of the physicians caring for children following heart surgery had training outside your country? If yes, please name the physician, the place where the training occurred and the length of time of the training.
4. How many physicians are available to participate in the post-operative care of the children?
5. Do you have physicians in the intensive care unit 24 hours/day? How many are there at night?
6. Do you have nurses experienced in the care of patients following heart surgery? Do any of them have experience in caring for children following heart surgery?
7. Please give the number of nurses who would be involved in the direct care of children following heart surgery.
8. Please give the number of nurses who would be available in general to help with the children but would need direction, supervision and education.
9. Do you have a separate department for Respiratory Care (technicians who provide respiratory care of the patients on ventilators or those in need of respiratory assistance)? If yes, how many individuals are in the department with experience in children?
10. Please give the working shift times (e.g. 7 a.m. to 7 p.m.) for the doctors and nurses who staff the intensive care unit.
11. What is the nursing staff/occupied bed ratio in the intensive care unit? Is this ratio the same regardless of the time of day?
12. How many and which individuals working in the intensive care unit speak English? Are translators available all hours of the day if language is an issue?

**Operating room: Infrastructure and hardware**

1. How many operating rooms are there for cardiac surgery? How many would be available for visiting team program in pediatric cardiac surgery?
2. Are the cardiac surgery operating rooms air-conditioned?
3. How many cardiopulmonary bypass machines does the hospital have? How many would be available for a visiting teams program?
4. Please give the manufacturer and model of each cardiopulmonary bypass machine.
5. How many heater/cooler units does the hospital have? Please give the manufacture and model of each unit? How many would be available for the visiting teams program?
6. Please give the manufacturer and model of the anesthesia machine(s) that would be in the operating room(s) that would be used for a visiting pediatric cardiac surgery program.
7. Please give the manufacturer and model of the invasive pressure monitors in the cardiac surgery operating rooms. Please list the different channels available on these monitors (e.g. 2 invasive pressures, pulse oximetry, EKG, temperature, etc.)
8. Does your hospital have an invasive pressure monitor with pulse oximetry capability that is used as a transport monitor during the transfer of the patient from the operating room to the intensive care unit?
9. Do the cardiac surgery operating rooms have water controlled heater/cooler blankets for the beds?
10. Does each operating room have infusion pumps and syringe pumps for the delivery of intravenous fluids and inotropic agents? Please give the number of units of each pump (infusion or syringe), manufacturer and model.
11. Are the oxygen and air supplies for the cardiopulmonary bypass machine separate from those for the anesthesia machine or are they shared?
12. Are the cardiopulmonary bypass machines capable of delivering anesthetic gases (e.g. isoflurane) to the cardiopulmonary circuits via a gas delivery system?
13. Do you have a separate room for the storage and preparation of the cardiopulmonary bypass machine and storage of the components of the cardiopulmonary bypass circuit?
14. Please give the size of the operating rooms by length and width.
15. What is the mechanism of suction (vacuum) in the operating room? Is it central or provided by individual machines? Please list the number of central suction (vacuum) outlets or individual machines that are available in each operating room.
16. Is the electrical supply in the operating rooms the same as in the rest of the hospital? How many outlets are available in the operating room? Are the outlets evenly distributed throughout the room, or concentrated in one area?
17. How many surgical overhead lights are there in the operating rooms (i.e. one main and one satellite or some other configuration)?
18. Do you have fiber optic headlights for the surgeon in each operating room?
19. Is the operating table controlled manually or electrically? Is the operating table capable of being moved into multiple positions while the operation is being performed?
20. Please give the manufacturer and model of the electro-cautery unit.
21. Do you have a defibrillator in each cardiac surgery operating room? Please give the manufacturer and model? Do you have pediatric internal paddles to attach to the defibrillator?
22. Do you have temporary pacemaker pulse generators in each cardiac surgery operating room? Please give the manufacturer and model.
23. What type of sternal saw do you use for primary sternotomy? Is it pneumatic, electric or manual? Please give the manufacturer and model.
24. Do you have a separate sternotomy saw for repeat sternotomies? If yes, please provide the manufacturer, model and whether it is pneumatic or electric.
25. Do you have an instrument tray specifically for pediatric cardiac procedures?
26. Do you have a method for preparing sterile ice or ice-slush?
27. Do you have an ACT machine for each cardiac surgery operating room? Please give the manufacturer and model of the ACT machine.
28. Do you have an autoclave in the operating room areas for quick re-sterilization of instruments?
29. What method of sterilization does your central sterilization use? Steam, ethylene oxide or other?
30. Do you have a satellite laboratory in the operating room or must all samples for analysis go to the central laboratory?

# Operating room: Personnel

1. How many cardiac surgeons work at your hospital?
2. Do any of the cardiac surgeons operate on children? If yes, please provide a list of the operations performed on children in the last 12 months and the names of the surgeons who performed the operations. If no, then would any of the adult heart surgeons be willing to work with the visiting pediatric cardiac surgery team? If yes, how many would be willing to work during a two-week program? Would these individuals be available to help every day?
3. Does the hospital have anesthesiologists who routinely provide cardiac anesthesia? If yes, how many? Do any of these anesthesiologists work with children? If yes, how many? Please provide the names of the anesthesiologists who would be willing to work with the visiting pediatric cardiac surgery team.
4. Does the hospital have nurses dedicated to working only in the heart surgery operating rooms? If yes, how many per operating room?
5. How many perfusionists work at the hospital? Do any of them have experience in pediatric perfusion? If yes, how many?
6. Are there technicians to help with anesthesia and perfusion services? If yes, how many per operating room?
7. Do any of the cardiac surgeons or cardiac anesthesiologists have educational experience outside of your country? If yes, who are the individuals, where did they train and for what period of time?
8. How many and which individuals in the operating room speak English? Are translators available all hours of the day if language is an issue?

**Cardiology services: Infrastructure and Hardware**

1. Does the hospital have a cardiac catheterization laboratory? If yes, please provide the manufacturer, model and whether it is a monoplane or biplane unit.
2. If you have a catheterization laboratory do you have any cardiologists who provide catheterizations to children? Does anyone provide interventional procedures in the laboratory? If yes, does anyone provide these services to children? If yes, please provide the name of the individual and a list of the interventional procedures he/she has performed on children in the last 12 months.
3. Does the hospital have a cardiac ECHO machine? If yes, how many machines, manufacturer and model please. If yes, please provide the sizes of the probes available and whether or not there is a pediatric trans-esophageal probe.
4. Does the hospital have pulse oximeters in either the catheterization laboratory or ECHO laboratory?

# Cardiology services: Personnel

1. Does the hospital have pediatric cardiologists? If yes, how many? If no, who provides the diagnosis of the children?
2. Will the cardiologists be willing to provide a list of candidates for surgery at least 1 month before the team arrives?
3. Is there a cardiologist in the hospital daily?
4. How many and which individuals who work in the cardiology areas speak English? Are translators available all hours of the day if language is an issue?

# Support services: Blood bank, clinical laboratory and radiology

1. Does the hospital have its own blood bank or is the blood bank located outside the hospital?
2. Will the team have access to blood products at any hour of the day?
3. What components are available? Whole blood, packed red blood cells, plasma, platlets, cryoprecipitate?
4. Are all the pre-operative patients screened for HIV?
5. Is the central laboratory available at all hours of the day? Can the laboratory provide the following measurements? Na, K, Cl, HCO3, glucose, ionized Ca, Mg, arterial blood gas analysis, hematocrit, hemoglobin, platelet count, partial thromboplastin time, INR, serum albumin.
6. Is there a satellite laboratory for the determination of arterial blood gases, blood counts and electrolytes? If yes, where is it located? If no, how long does it take to receive results for samples taken in the operating rooms or intensive care unit?
7. Does radiology provide portable x-rays for patients in the intensive care unit? If yes, how long does it take to obtain and then receive the x-ray? Are these services available all hours of the day?

**Pharmacy:**

1. Does your pharmacy prepare your cardioplegia solution? If yes, please provide us with the ingredients and concentrations that you use. If no, please provide us with the name of the commercially prepared cardioplegia solution that you use.
2. Does the hospital have only one pharmacy or do you have a satellite pharmacy in addition to the central pharmacy? Is the pharmacy open all hours of the day?
3. Does the pharmacy prepare the inotropic infusions for the patients or is this performed in the intensive care unit and operating rooms as needed?
4. Does the pharmacy have the capability of providing intravenous parenteral nutrition for children?
